# Familial *NEDD4L* variant in periventricular nodular heterotopia and in a fetus with hypokinesia and flexion contractures

**DOI:** 10.1002/mgg3.490

**Published:** 2018-11-04

**Authors:** Miriam Elbracht, Florian Kraft, Matthias Begemann, Petra Holschbach, Michael Mull, Ildiko M. Kabat, Britta Müller, Martin Häusler, Ingo Kurth, Ute Hehr

**Affiliations:** ^1^ Institute of Human Genetics, Medical Faculty RWTH Aachen University Aachen Germany; ^2^ Division of Neuropediatrics and Social Pediatrics, Department of Pediatrics, Medical Faculty RWTH Aachen University Aachen Germany; ^3^ Department of Diagnostic and Interventional Neuroradiology, Medical Faculty RWTH Aachen University Aachen Germany; ^4^ Department of Radiology, Radiologie Universität Bonn Ildiko M. Kabat University Hospital Bonn Bonn Germany; ^5^ Department of Human Genetics, Medical Center University of Regensburg Regensburg Germany

**Keywords:** arthrogryposis, fetal hypokinesia, nanopore sequencing, *NEDD4L*, periventricular nodular heterotopias

## Abstract

**Background:**

Mutations in the HECT domain of *NEDD4L* have recently been identified in a cohort of eight patients with a syndromic form of bilateral periventricular nodular heterotopia (PVNH) in association with neurodevelopmental delay, cleft palate, and toe syndactyly (PVNH7).

**Methods:**

Case report based on NGS sequencing.

**Results:**

Here, we describe a girl with a novel heterozygous *NEDD4L* missense variant, p.Tyr679His, and characteristic clinical findings, including bilateral periventricular nodular heterotopia, cleft palate and mild toe syndactyly. Molecular testing from peripheral blood identified the healthy father to carry the *NEDD4L* variant in mosaic state. Notably, a previous pregnancy of the couple had been terminated due to a complex fetal developmental disorder, including hypokinesia and flexion contractures. Upon review, this affected fetus was also shown to carry the familial *NEDD4L* variant.

**Conclusion:**

Our findings may suggest a broader spectrum of *NEDD4L*‐associated phenotypes, including severe prenatal neurodevelopmental manifestations, which might represent yet another genetic form of fetal hypokinesia with flexion contractures.

## 1 INTRODUCTION

NEDD4L (ubiquitin protein ligase NEDD4‐like), also known as NEDD4‐2 (neural precursor cell expressed developmentally down‐regulated 4‐like), is a regulatory protein involved in the differentiation and function of the central nervous system as well as of embryonal mesenchymal structures. NEDD4L has been shown to regulate the function of neuronal voltage‐gated sodium channels especially in the fetal central nervous system, and NEDD4L depletion causes neural tube closure defects in Xenopus embryos (Ekberg et al., 2014; Zhang, Ding, Chen, & Tao, 2014). It has also been associated with the pathogenesis of salt‐sensitive hypertension (Rizzo & Staub, 2015) and mesenchymal differentiation (Qu et al., 2016).

Heterozygous mutations in the *NEDD4L* gene have recently been identified to cause a novel syndromic form of periventricular nodular heterotopia in humans (PVNH7; OMIM #617201) (Broix et al., 2016). PVNH describes the nodular accumulation of neuronal precursor cells at the site of their origin along the lateral walls of both lateral ventricles, most likely resulting from failed initiation of neuronal migration (Ferland & Guerrini, 2009; Lange et al., 2015). So far, eight patients (five females, three males) with autosomal dominant *NEDD4L* mutations have been described (Broix et al., 2016; Kato et al., 2017), showing bilateral PVNH in combination with syndactyly of toes 2–3 and cleft palate or bifid uvula in seven patients. All patients presented with moderate‐to‐severe developmental delay. Two patients are sibs, and their unaffected mother was shown to be mosaic carrier for a heterozygous *NEDD4L* missense mutation (Broix et al., 2016). The youngest patient with a molecularly confirmed *NEDD4L* disorder was a 4‐month‐old male described by Broix et al.; the oldest patient was diagnosed at the age of 12 years and developed late‐onset seizures and severe intellectual disability. So far, no data on the long‐term course and prognosis of patients with *NEDD4L* mutations are available. All reported patients are carriers of a heterozygous missense mutation in the HECT domain of *NEDD4L* which regulates protein ubiquitination. Functional studies indicate that mutant NEDD4L leads to conformational changes in the protein and constitutive activation of the catalytic domain which can trigger autoubiquitination and protein degradation (Escobedo et al., 2014).

**Figure 1:**
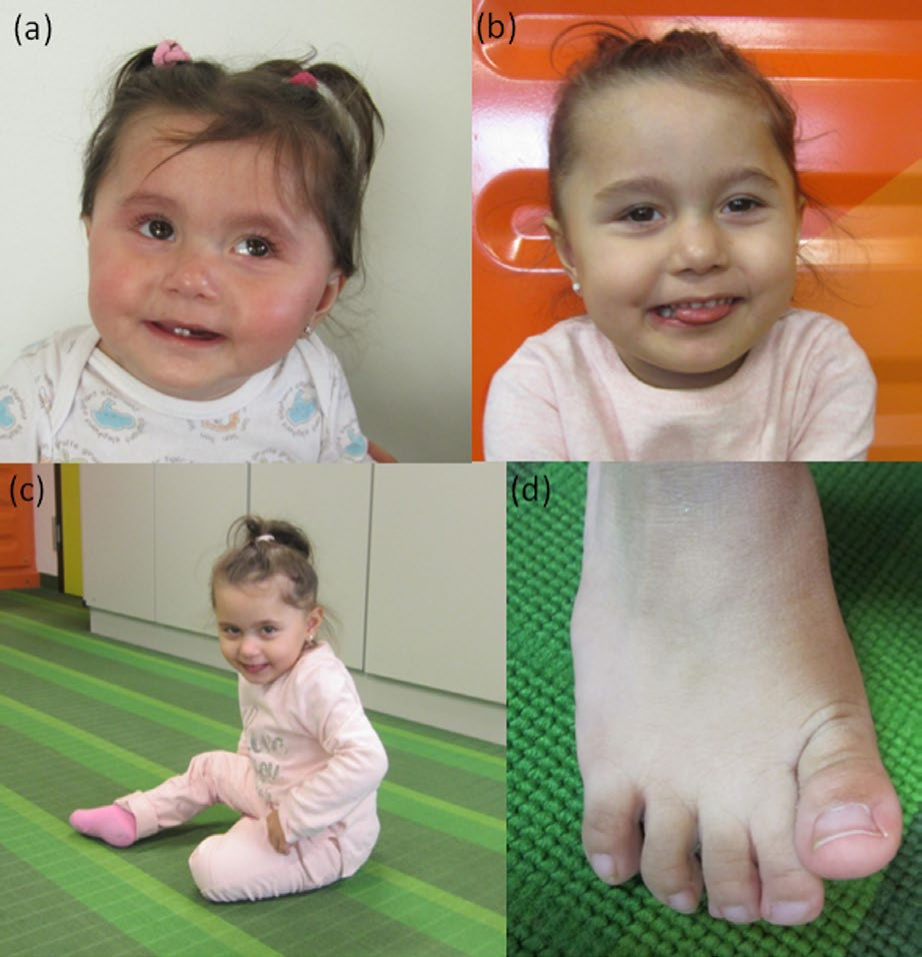
Index patient: (a) at the age of 17 months, (b–d) at the age of 2 years and 9 months/12 years; (a,b) facial aspect without marked dysmorphism, but mild strabismus, (c) sitting position from which she can move ahead, (d) mild second/third toe syndactyly of the patient

Here, we describe a girl with cleft palate, mild toe syndactyly 2–3 and severe developmental delay (Figure 1). Brain magnetic resonance imaging (MRI) findings of PVNH suggested a neuronal migration disorder, and a *NEDD4L* variant was identified in line with her clinical presentation. Mosaicism for the variant was detected in peripheral lymphocytes of her father. Upon review, this *NEDD4L* variant was also detected in fetal tissue of a previous pregnancy of the couple, terminated because of fetal hypokinesia, flexion contractures of hands, and mild retrogenia, suggesting a broader *NEDD4L* phenotypic spectrum including severe prenatal neurodevelopmental manifestation.

## 2 RESULTS

Our patient is the second child of healthy non‐consanguineous parents from Serbia and Macedonia, respectively. Pregnancy was uneventful; however, amniocentesis was requested in the 16th gestational week by the 29‐year‐old mother and detected an unexpected monosomy X in 20% of the analyzed cells (karyotype 45,X/46,XX), in the absence of any pathologic fetal ultrasound findings. The girl was born at term with short stature (46 cm; 1st percentile) but normal birthweight (3,230 g, 24th percentile) and head circumference (33 cm, 6th percentile). Newborn screen was negative. Clefting of the palate led to feeding difficulties and required surgical intervention at the age of 8 months. Bilateral mild syndactyly of the second and third toes was noted.

At the age of 11 months, developmental delay became obvious prompting further investigations. By this time, the girl showed a small head circumference (3rd–10th percentile; 43.5 cm), normal length (75 cm, 50th percentile) and weight (8,550 g, 10th percentile), adducted thumbs, and truncal hypotonia; she could neither turn from back to front, nor sit unsupported. She had a low posterior hairline, a short neck, a bilateral sandal gap, and mild plagiocephaly. EEG abnormalities of spikes and sharp waves were identified in the absence of clinically recognizable seizures. Deep tendon reflexes were hyperreactive with elevated reflex zones and reactive cloni. In contrast to truncal hypotonia, the girl showed bilateral talipes equinovarus and muscular hypertonia on both legs. Urine and blood investigations for metabolic disorders showed normal results.

At the age of 2 years and 9 months, the girl's height and weight were still within the normal range. Her head circumference was on the third percentile (47 cm). She could not speak but was able to communicate with sounds and gestures. She could sit and move along in a sitting position (Figure 1a–d). She did not crawl, but was able to make first steps with help.

**Figure 2:**
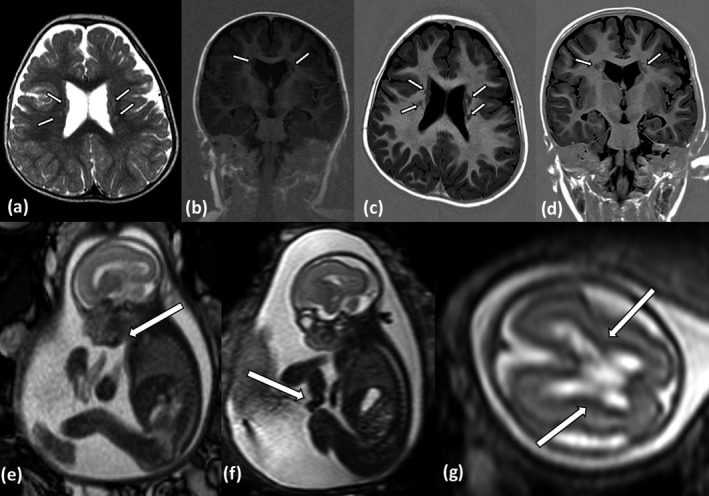
Index patient: (a,b) cranial MRI at the age of 17 months, (a) T2 axial, (b) T1 IR coronal; (c,d) Cranial MRI at the age of 2 9/12 years, (c) T1 IR axial, (d) T1 IR coronal. MRI follow‐up shows bilateral subependymal heterotopia with gray matter nodules, as well as deep subcortical heterotopias. The cortex shows areas of excessive infolding and irregularity on both sides which represent a cortical malformation. Fetus of the prior pregnancy: (e–g) MRI findings at the age 23 + 6 gestational week, T2w images. (e) Retrognathia; (f) flexion contracture of the hand; (g) mild periventricular hyperintensity

At the age of 17 months, cranial magnetic resonance imaging (MRI) was carried out and was consistent with MRI findings at the age of 2 years and 9 months. Spinal studies were included because of mild progressive lower limb spasticity. Imaging confirmed bilateral subependymal periventricular nodular heterotopia (Figure 2a–d) without spinal pathology, as well as deep subcortical heterotopias which are not seen in *FLNA‐* or other related PVNH disorders. The axial T2 and T1 images showed areas of excessive cortical infolding with mildly thick and irregular cortex in the posterior frontal–anterior perisylvian regions on both sides. These abnormalities represent a cortical malformation, most likely polymicrogyria. The hippocampus of our patient appeared to be normal. Cortical malformations in addition to the PVNH are also discussed in the MRI findings of the patients of Broix et al. and Kato et al. (Broix et al., 2016; Kato et al., 2017). Written informed consent was obtained from the study participants after approval from the Institutional Review Boards at the participating institution (Uniklinik RWTH Aachen: EK302‐16). Consent was obtained according to the Declaration of Helsinki, and consent was given for the publication of patient photographs.

Mutation screening for PVNH was performed by massive parallel sequencing (Illumina Nextera enrichment, 150‐bp pair run on a NextSeq 500 platform, Illumina, bioinformatics assessment using an in‐house pipeline) to investigate the known PVNH‐associated genes *ARFGEF2, FLNA, TUBA1A,* and the more recently published *NEDD4L* gene. The analysis revealed a heterozygous missense variant, c.2035T>C (p.Tyr679His) within the HECT domain of *NEDD4L* (NM_001144967.2; coverage average 93.41‐fold). This novel missense variant affects a previously reported codon, p.Tyr679Cys (Broix et al., 2016). The variant is predicted to be “likely pathogenic” accordingly to the ACMG standards and bioinformatic prediction programs (Mutation taster 2, SIFT, Polyphen 2, CADD Phred).

**Figure 3:**
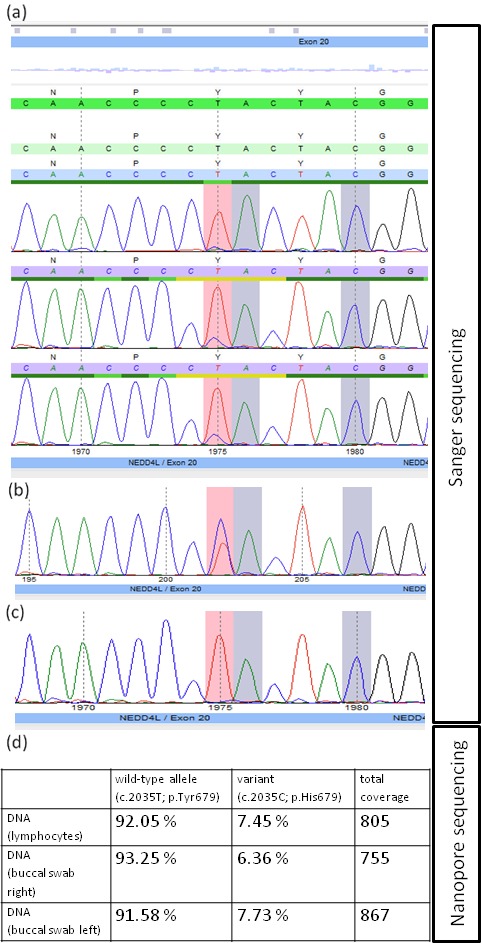
Electropherograms of the identified *NEDD4L* missense variant (highlighted by pink boxes) in the family: father (a, forward‐, reverse‐, and confirmation reverse‐primer), index case (b, reverse primer), mother (c, reverse primer). Note low‐level mosaicism for the c.2035T>C (p.Tyr679His) as detected by Sanger sequencing. (d) Percentage of wild‐type and mutant alleles in different tissues of the father by high‐coverage nanopore sequencing

Parental carrier testing did not show the respective variant in the maternal peripheral blood, but revealed paternal mosaicism for this *NEDD4L* variant in blood cells. Mosaicism in paternal blood cells has been assessed by Sanger sequencing and indicated low‐grade mosaicism for the variant in the blood sample of the father (Figure 3a–c). However, Sanger sequencing is a qualitative measure, and thus, we decided to analyze the DNA of the father by next‐generation sequencing. We have therefore chosen long‐read nanopore sequencing (Oxford Nanopore Technologies) and sequenced PCR products spanning the variant c.2035T>C (p.Tyr679His) with high coverage (Figure 3d). Indeed, deep nanopore sequencing revealed a mosaicism of approximately 10% in blood cells as well as in cells from buccal swaps, corroborating a somatic mosaicism in the father.

The father reported mild learning difficulties in school, but no neurological or cognitive features of *NEDD4L‐*associated PVNH7 had been observed previously or at the time of examination. He had no cleft palate or cleft uvula, a normal head circumference (56 cm, 30th percentile). After identification of paternal mosaicism for the pathogenic *NEDD4L* variant, the family history was reevaluated. The index girl has an older healthy brother. However, a second pregnancy had been terminated in the 26th gestational week because of complex fetal sonographic findings, including fetal hypokinesia, permanent flexion contractures of elbows and hands, mild retrogenia, and mild thickening of the cavum septi pellucidi, subsequently confirmed by fetal MRI (Figure 2e–g). No further brain anomalies were observed in the fetal MRI, and a postmortem examination of the fetus had been declined by the parents. Chromosomal analysis after amniocentesis showed a normal fetal male karyotype. Fetal DNA from the amniocentesis was subsequently analyzed and revealed the familial heterozygous *NEDD4L* missense variant p.Tyr679His; however, the parents did not consent to further genetic testing such as whole‐exome sequencing.

## 3 DISCUSSION

With the identification of heterozygous mutations in the HECT domain of *NEDD4L,* an additional genetic form of periventricular nodular heterotopia has been identified (Broix et al., 2016). Similar to the present case, all of the previously described eight patients showed developmental delay including two individuals with severe intellectual disability. Interestingly, clefting of the palate and syndactyly of the toes 2–3 appear to be quite consistent features, indicating a broader role of the *NEDD4L* gene product beyond initiation of neuronal migration.

Clinical findings in our patient are in line with the index patients described by Broix et al. and the patient by Kato et al. Despite her marked developmental disorder, she shows very good non‐verbal communication skills. EEG recordings showed abnormalities; however, she did not develop epilepsy to date (age 3). Spinal cord pathology has been evaluated because of truncal hypotonia and lower limb spasticity with talipes equinovarus but could not be confirmed by spinal MRI. Together with the observed contractures of the fetus and the heterozygous *NEDD4L* variant, our observations suggest a much broader clinical spectrum.

At the time of examination, the index girl did not show any clinical findings indicative for the prenatally detected mosaic monosomy X. Postnatal confirmation of the molecular findings has been offered but have been declined by the parents.

Six of the previously described eight patients are carriers of a de novo mutation in *NEDD4L*. Our report adds a second family with parental germline mosaicism and transmission of the *NEDD4L* variant to two affected offspring.

It seems reasonable that the *NEDD4L* variant was causative for the fetal hypokinesia syndrome with flexion contractures in the second pregnancy of the couple (Figure 2e,f). The patient DDDP110533 described in Broix et al. (2016), who carried another missense mutation at the same affected codon as our family, was reported to show arthrogryposis; thus, both observations point toward a broader *NEDD4L* phenotypic spectrum than currently anticipated. Retrospectively one might discuss whether the lateral walls of both lateral ventricles might show a slightly pronounced echogenicity on fetal MRI comparable to the cortical zone (Figure 2g). At the time of examination (23 + 6 gestational week), however, no nodules were observed. In general, prenatal diagnosis of periventricular nodular heterotopia by ultrasound or fetal MRI is often missed, especially in cases without prior clinical suspicion or complex brain malformations (Blondiaux et al., 2013; Sahinoglu, Yapicier, & Ozcan, 2016). A postmortem macroscopic or histological examination of the fetus had not been carried out. Moreover, the differential diagnosis of fetal hypokinesia and flexion contractures is complex and includes arthrogryposis syndromes, neuromuscular diseases, and several developmental disorders, which have not been ruled out formally for the previous pregnancy. The fetus had a normal male karyotype, and no obvious further cerebral or extracerebral malformations were detected.

Our data confirm an important role for heterozygous *NEDD4L* variants in the pathogenesis of PVNH combined with a more complex brain malformation and a broad phenotypic spectrum. The consistently observed syndromic features of cleft palate and syndactyly 2–3 emerge as characteristic hallmarks in the differential diagnosis to *FLNA*‐associated PVNH. The observation of parental mosaicism in two out of seven families with *NEDD4L*‐associated PVNH warrants caution and should be specifically addressed in parental carrier testing and genetic counseling. This is in line with other reports which show that parental mosaicism with increased recurrence risk is more frequent than previously estimated (Heinzen et al., 2018; Myers et al., 2018).

## CONFLICT OF INTEREST

The authors declare no conflict of interest.

## References

[mgg3490-bib-0001] Blondiaux, E. , Sileo, C. , Nahama‐Allouche, C. , Moutard, M. L. , Gelot, A. , Jouannic, J. M. , … Garel, C. (2013). Periventricular nodular heterotopia on prenatal ultrasound and magnetic resonance imaging. Ultrasound in Obstetrics and Gynecology, 42(2), 149–155. 10.1002/uog.12340 23151899

[mgg3490-bib-0002] Broix, L. , Jagline, H. , Ivanova, E. , Schmucker, S. , Drouot, N. , Clayton‐Smith, J. , … Chelly, J. (2016). Mutations in the HECT domain of NEDD4L lead to AKT‐mTOR pathway deregulation and cause periventricular nodular heterotopia. Nature Genetics, 48(11), 1349–1358. 10.1038/ng.3676 27694961 PMC5086093

[mgg3490-bib-0003] Ekberg, J. A. , Boase, N. A. , Rychkov, G. , Manning, J. , Poronnik, P. , & Kumar, S. (2014). Nedd4‐2 (NEDD4L) controls intracellular Na(+)‐mediated activity of voltage‐gated sodium channels in primary cortical neurons. The Biochemical Journal, 457(1), 27–31. 10.1042/BJ20131275 24152020

[mgg3490-bib-0004] Escobedo, A. , Gomes, T. , Aragon, E. , Martin‐Malpartida, P. , Ruiz, L. , & Macias, M. J. (2014). Structural basis of the activation and degradation mechanisms of the E3 ubiquitin ligase Nedd4L. Structure, 22(10), 1446–1457. 10.1016/j.str.2014.08.016 25295397

[mgg3490-bib-0005] Ferland, R. J. , & Guerrini, R. (2009). Nodular heterotopia is built upon layers. Neurology, 73(10), 742–743. 10.1212/WNL.0b013e3181b529b1 19625704

[mgg3490-bib-0006] Heinzen, E. L. , O'Neill, A. C. , Zhu, X. , Allen, A. S. , Bahlo, M. , Chelly, J. , … Epilepsy Phenome/Genome Project (2018). De novo and inherited private variants in MAP1B in periventricular nodular heterotopia. PLoS Genetics, 14(5), e1007281 10.1371/journal.pgen.1007281 29738522 PMC5965900

[mgg3490-bib-0007] Kato, K. , Miya, F. , Hori, I. , Ieda, D. , Ohashi, K. , Negishi, Y. , … Saitoh, S. (2017). A novel missense mutation in the HECT domain of NEDD4L identified in a girl with periventricular nodular heterotopia, polymicrogyria and cleft palate. Journal of Human Genetics, 62(9), 861–863. 10.1038/jhg.2017.53 28515470

[mgg3490-bib-0008] Lange, M. , Kasper, B. , Bohring, A. , Rutsch, F. , Kluger, G. , Hoffjan, S. , … Hehr, U. (2015). 47 patients with FLNA associated periventricular nodular heterotopia. Orphanet Journal of Rare Diseases, 10, 134 10.1186/s13023-015-0331-9 26471271 PMC4608144

[mgg3490-bib-0009] Myers, C. T. , Hollingsworth, G. , Muir, A. M. , Schneider, A. L. , Thuesmunn, Z. , Knupp, A. , … Mefford, H. C. (2018). Parental mosaicism in "de novo" epileptic encephalopathies. New England Journal of Medicine, 378(17), 1646–1648. 10.1056/NEJMc1714579 29694806 PMC5966016

[mgg3490-bib-0010] Qu, M. H. , Han, C. , Srivastava, A. K. , Cui, T. , Zou, N. , Gao, Z. Q. , & Wang, Q. E. (2016). miR‐93 promotes TGF‐beta‐induced epithelial‐to‐mesenchymal transition through downregulation of NEDD4L in lung cancer cells. Tumour Biology, 37(4), 5645–5651. 10.1007/s13277-015-4328-8 26581907 PMC6528179

[mgg3490-bib-0011] Rizzo, F. , & Staub, O. (2015). NEDD4‐2 and salt‐sensitive hypertension. Current Opinion in Nephrology and Hypertension, 24(2), 111–116. 10.1097/MNH.0000000000000097 25602517

[mgg3490-bib-0012] Sahinoglu, Z. , Yapicier, O. , & Ozcan, N. (2016). Prenatal diagnosis of periventricular nodular heterotopia in borderline ventriculomegaly using sonography and magnetic resonance imaging. Journal of Clinical Ultrasound, 44(8), 510–513. 10.1002/jcu.22350 26990213

[mgg3490-bib-0013] Zhang, Y. , Ding, Y. , Chen, Y. G. , & Tao, Q. (2014). NEDD4L regulates convergent extension movements in Xenopus embryos via Disheveled‐mediated non‐canonical Wnt signaling. Developmental Biology, 392(1), 15–25. 10.1016/j.ydbio.2014.05.003 24833518

